# Deep reinforced learning heuristic tested on spin-glass ground states: The larger picture

**DOI:** 10.1038/s41467-023-41106-y

**Published:** 2023-09-14

**Authors:** Stefan Boettcher

**Affiliations:** Department of Physics, Emory University, Atlanta, GA 30322 USA

**Keywords:** Complex networks, Glasses, Computational science

**arising from** C. Fan et al. *Nature Communications* 10.1038/s41467-023-36363-w (2023)

In ref. ^1^, the authors present a deep reinforced learning approach to augment combinatorial optimization heuristics. In particular, they present results for several spin glass ground state problems^2^, for which instances on non-planar networks are generally NP-hard, in comparison with several Monte Carlo-based methods, such as simulated annealing (SA) or parallel tempering (PT)^3^. Here, we examine those results in the context of well-established literature and find that, albeit fast and capable for small instance sizes, the presentation lacks signs of the claimed superiority for larger instances, unless one competes with Greedy Search for speed.

Indeed, the results of ref. ^1^ demonstrates that the reinforced learning improves the results over those obtained with SA or PT, or at least allows for reduced runtimes for the heuristics before results of comparable quality have been obtained relative to those other methods. To facilitate the conclusion that their method is “superior”, the authors of ref. ^1^ pursue two basic strategies: (1) A commercial GUROBI solver (see https://www.gurobi.com/) is called on to procure a sample of exact ground states as a testbed to compare with, and (2) a head-to-head comparison between the heuristics is given for a sample of larger instances where exact ground states are hard to ascertain. Here, we put these studies into a larger context, showing that the claimed superiority is at best marginal for smaller samples and becomes essentially irrelevant with respect to any sensible approximation of true ground states in the larger samples. For example, this method becomes irrelevant as a means to determine stiffness exponents *θ* in *d* > 2, as mentioned by the authors, where the problem is not only NP-hard but requires the subtraction of two almost equal ground-state energies and systemic errors in each of ≈ 1% found here are unacceptable^4^. This larger picture of the method arises from a straightforward finite-size corrections study over the spin glass ensembles the authors employ, using data that has been available for decades^5,6^.

In our investigation here, we focus on mainly two ensembles of NP-hard problems the authors utilize: The Edwards–Anderson spin glass on a cubic lattice (EA in *d* = 3) with periodic boundary conditions^7^ and the mean-field (all-to-all connected) Sherrington–Kirkpatrick spin glass (SK)^8^. The ensemble for both models consists of instances where all bonds are chosen randomly from a normal distribution of zero mean and unit variance. The ensemble is parametrized by its size, i.e., the number of variables *N* in a spin configuration $\overrightarrow{\sigma }$, where *N* = *L*^3^ in the case of EA. With those hard combinatorial problems, there are many ways to find exact solutions for instances of small *N*, such as a solver like GUROBI, however, for any practical application at large *N*, the super-polynomial rise in complexity necessitates the use of heuristic methods. Thus, the scalability of a heuristic is of particular concern. In the formal study of computational complexity, this is typically addressed by establishing bounds on an all-encompassing worst-case scenario^9^. For many complicated meta-heuristics^10^, such as the case of the method presented here, insights into the capability of a heuristic can be gained only from comparative studies over widely accepted testbeds of instances or those selected from specific ensembles. The authors have clearly adopted the ensemble approach^1^.

Especially with regard to scaleability, the ensemble picture deserves particular attention, for the following reasons. Those ensembles typically have a “thermodynamic limit”, i.e., their averages are well-defined and possess a clear meaning for *N* → *∞*, which is a typical large instance approach. At times, that limit may even be solvable, such as in the case of SK^11^, but that is not essential here, as exemplified by EA. More importantly, that limit is usually attained in an equally well-defined manner through finite-size corrections (FSC). To be specific in this context, for the cost function a heuristic is trying to minimize, the authors have chosen the ground state energy density, ${e}_{0}=\mathop{\min }\nolimits_{\overrightarrow{\sigma }}H\left(\overrightarrow{\sigma }\right)/N$, of the Hamiltonian *H* for each of their (physically motivated) spin glass ensembles. Instances are generated via random choices of bonds *J*_*ij*_ from a characteristic distribution *P*(*J*), see Eq. (1) in ref. ^1^. If the thermodynamic limit for the ensemble-averaged ground-state energy density ${\left\langle {e}_{0}\right\rangle }_{N=\infty }$ exists, FSC assumes the asymptotic scaling form

1 $${\left\langle {e}_{0}\right\rangle }_{N} \sim {\left\langle {e}_{0}\right\rangle }_{N=\infty }+\frac{A}{{N}^{\omega }}+\ldots,\qquad (N\to \infty ),$$

for a constant *A* and a correction exponent *ω*(>0). Clearly, other forms of corrections might exist and higher-order terms could well obscure the assumed behavior deep into the large-*N* regime. Yet, self-consistency with the form in Eq. (1) of the actual data for small *N*, where reliable (or exact) results can be ascertained, often provides a powerful baseline to assess the scalability of a heuristic^12,13^. This is certainly the case here, and it provides a larger picture of the results in ref. ^1^.

Long before the PT results^3^ that the authors reference in their study of EA in *d* = 3, virtually identical results have been found by Pal^5^ using a genetic algorithm (GA). Despite the doubts the authors raise (in the caption (Note that several references in ref. ^1^ are incorrect, e.g., in the caption to Fig. 5 “ref. 51” should be to ref. 50 and the label “f” should be “d” for the 3*d*-EA at *L* = 10.) of their Fig. 5), both the PT and the GA data exhibit a consistent scaling picture, shown here in Fig. 1. While the authors do not provide any tabulated data for their corresponding results, at least for the larger samples we can extract estimated values for their best results (for DIRAC-SA, shown as red circles in Fig. 1) from the plots provided in their Fig. S5d–f. There, the fact that the DIRAC-SA data is better than either PT or SA is taken as evidence of the superiority of their method by the authors. However, considering how far separated from any actual ground states every one of the datasets employed in this comparison really is, this advantage, whether in speed or in accuracy, is rather inconsequential in the larger picture of Fig. 1.

**Fig. 1:**
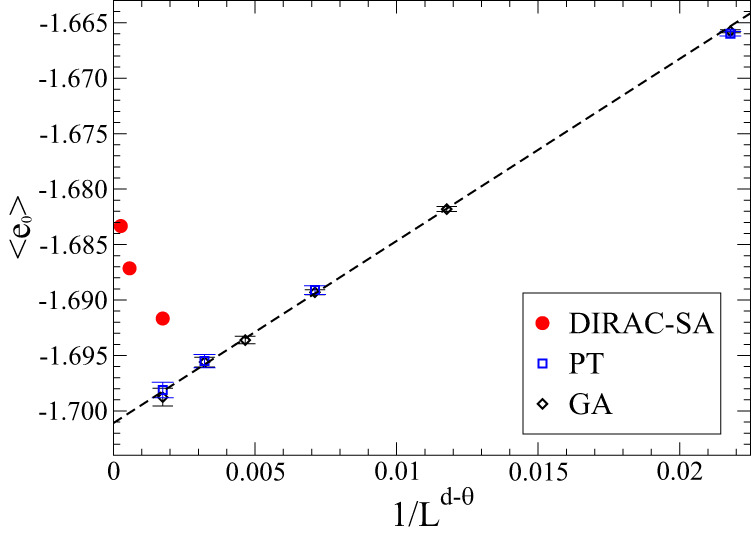
Extrapolation plot according to the finite-size corrections form in Eq. (1) for the ensemble-averaged ground state energy densities obtained with various heuristics for EA in *d* = 3. Previous data obtained with GA^5^ or PT^3^ for a range of system sizes *N* = *L*^*d*^ up to *L* = 14 exhibits a consistent asymptotic scaling with corrections ~ 1/*N*^*ω*^ and *ω* = 1 −*θ*/*d* ≈ 0.92, as discussed in ref. ^12^. The linear fit (dashed line) with *x* = 1/*L*^2.76^ has the form ${\left\langle {e}_{0}\right\rangle }_{N=\infty }+Ax$ with ${\left\langle {e}_{0}\right\rangle }_{N=\infty }\approx -1.701$ and *A* ≈ 1.641. The corresponding data for *L* = 10, 15, 20 from ref. ^1^ (red circles) diverge increasingly from the expected values for typical ground states.

Similarly, the results the authors provide for SK prove inconclusive in the larger picture of long-established results for this case^6,13,14^. Here, ref. ^1^ merely provides results of their method for quite small instances, where GUROBI allows to obtain exact ground states for comparison. While these results are indeed consistent with the predicted scaling, as shown in Fig. 2, the sizes bounded by *N* ≤ 216 considered in their study have very limited predictive power about the scalability of their method for any size that would make their method competitive, either in speed or in accuracy, with state-of-the-art heuristics at larger *N*. After all, with an ensemble approach, it is not necessary to rely on exactly solved instances to make impactful comparisons, as our discussion of EA demonstrates.

**Fig. 2:**
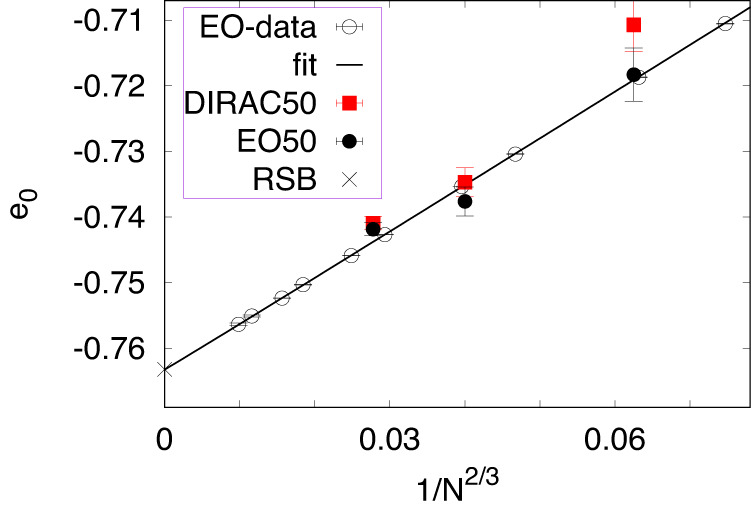
Extrapolation plot of ensemble-averaged ground state energy densities for SK according to Eq. (1) with *ω* = 2/3. For SK, theory (RSB^11^) predicts an exact result for the limit *N* → *∞*, ${\left\langle {e}_{0}\right\rangle }_{N=\infty }=-0.7632\ldots$, marked by ×. The reference data (open circles) for up to *N* = 1023, averaged over at least 10^5^ instances each, was obtained with the extremal optimization heuristic (EO)^6^. That the asymptotic fit (line) of this data predicts ${\left\langle {e}_{0}\right\rangle }_{N=\infty }$ with high accuracy adds confidence in the scaling. The data for 50 instances each at *N* = 64, 125, and 216 from ref. ^1^ (red squares) matches within errors to a similar random sample of 50 instances each optimized with EO (filled circles). Note that the ground-state energy variances for SK are typically broader than standard deviations^6^. The DIRAC50 data here was obtained from Fig. S15d–f in ref. ^1^, which required the addition division by $\sqrt{N}$ in the ground state energy densities when a univariate ($\left\langle {J}^{2}\right\rangle=1$) bond distribution is used, i.e., ${\left\langle {e}_{0}\right\rangle }_{N=64}=-5.6856/\sqrt{64}=-0.7107$, ${\left\langle {e}_{0}\right\rangle }_{N=125}=-8.2141/\sqrt{125}=-0.7347$, and ${\left\langle {e}_{0}\right\rangle }_{N=216}=-10.8895/\sqrt{216}=-0.7409$.

In conclusion, a comparison with existing data shows little evidence for the claimed superiority of the deep reinforcement learning strategy to enhance optimization heuristics proposed in ref. ^1^. The comparison provided here for both, a sparse short-range and a dense infinite-range spin glass model, is quite exemplary for all the ensembles the authors discuss so that this conclusion is likely not particular to these two cases. The authors should be lauded for having demonstrated some gains relative to simple greedy algorithms for EA^15^, but their results remain too far from optimality, even if under the < 1% level we found in Fig. 1, to be of any use in applications to the physics of spin glasses the authors imply. For example, in the stiffness problem, one determines the ground state of an instance in EA and again for reversed boundary conditions, which inserts a relative domain wall between the ground states with separate energies ${e}_{0}^{1,2}(L) \sim {\left\langle {e}_{0}\right\rangle }_{L=\infty }+{A}_{1,2}/{L}^{d\omega }+\ldots$. That domain wall has a much smaller energy, ${{{{{{{\rm{{{\Delta }}}}}}}}}}e=\left|{e}_{1}-{e}_{2}\right|\sim {{{{{{{\rm{{{\Delta }}}}}}}}}}A/{L}^{d\omega }\to 0$, which relates FSC to the stiffness exponent via *dω* = *d* − *θ*^12^, as used in Fig. 1. These exponents were determined for EA in dimensions *d* = 3, …, 7 by finding ground states for millions of dilute lattices with up to *N* = 10^7^ using a hybrid EO algorithm^4,16^. Hence, the heuristics chosen as a base for their comparison are surprisingly narrow, considering that the authors refer to ref. ^2^ for the use of heuristics for spin glasses, which also discusses GA and EO.

## Data Availability

Most data discussed in this comment is already available directly from the respective references cited. Beyond that, any data presented or discussed in this comment is also available on request from the author via email.
